# Type 2 Diabetes Mellitus and Altered Immune System Leading to Susceptibility to Pathogens, Especially *Mycobacterium tuberculosis*

**DOI:** 10.3390/jcm8122219

**Published:** 2019-12-16

**Authors:** Steve Ferlita, Aram Yegiazaryan, Navid Noori, Gagandeep Lal, Timothy Nguyen, Kimberly To, Vishwanath Venketaraman

**Affiliations:** 1 College of Osteopathic Medicine of the Pacific, Western University of Health Sciences, Pomona, CA 91766-1854, USA; steve.ferlita@westernu.edu (S.F.); nnoori@westernu.edu (N.N.); gagandeep.lal@westernu.edu (G.L.); timothy.nguyen@westernu.edu (T.N.); 2 Graduate College of Biomedical Sciences, Western University of Health Sciences, Pomona, CA 91766-1854, USA; aram.yegiazaryan@westernu.edu; 3 Department of Basic Medical Sciences, College of Osteopathic Medicine of the Pacific, Western University of Health Sciences, Pomona, CA 91766-1854, USA; kimberly.to@westernu.edu

**Keywords:** type 2 diabetes, co-morbidities, co-infections, cytokines, inflammation, redox imbalance, antioxidants

## Abstract

There has been an alarming increase in the incidence of Type 2 Diabetes Mellitus (T2DM) worldwide. Uncontrolled T2DM can lead to alterations in the immune system, increasing the risk of susceptibility to infections such as *Mycobacterium tuberculosis (M. tb)*. Altered immune responses could be attributed to factors such as the elevated glucose concentration, leading to the production of Advanced Glycation End products (AGE) and the constant inflammation, associated with T2DM. This production of AGE leads to the generation of reactive oxygen species (ROS), the use of the reduced form of nicotinamide adenine dinucleotide phosphate (NADPH) via the Polyol pathway, and overall diminished levels of glutathione (GSH) and GSH-producing enzymes in T2DM patients, which alters the cytokine profile and changes the immune responses within these patients. Thus, an understanding of the intricate pathways responsible for the pathogenesis and complications in T2DM, and the development of strategies to enhance the immune system, are both urgently needed to prevent co-infections and co-morbidities in individuals with T2DM.

## 1. Introduction

### 1.1. Type 2 Diabetes Mellitus

Type 2 Diabetes Mellitus is a prevalent disease throughout the world. The World Health Organization (WHO) reports that an estimated 422 million people all over the world are living with diabetes. The WHO projects that diabetes will be the seventh leading cause of death in 2030 [1]. In the United States, the Center for Disease Control (CDC) reports that 30.3 million people have diabetes, with approximately 23.1 million people diagnosed every year and a staggering 84.1 million Americans in pre-diabetic stage [2]. Tuberculosis (TB) is a serious threat for people living with diabetes. In 2017, 20% of people with TB in the United States also had diabetes. Currently, one third of the population of the world is infected with latent *Mycobacterium tuberculosis (M. tb)* [3]. At this stage, the individual is not infectious; however, TB can be reactivated as a result of granuloma liquefaction due to immunodeficiency or immunocompromisation [4], as in the case with individuals suffering from Type 2 Diabetes Mellitus (T2DM) [5]. Due to the high prevalence of diabetes worldwide, necessary measures must be taken to understand, prevent, and treat T2DM.

Insulin, a peptide hormone produced by beta cells within the pancreas, facilitates the absorption of glucose into cells from blood, in order to maintain proper glucose levels [6]. T2DM is a disease characterized by the inability of the body to produce sufficient insulin, or the development of insulin resistance [7]. As a result, T2DM’s state of insufficient insulin production or insulin-resistance can have detrimental complications, including macrovascular diseases such as hypertension, coronary artery disease, heart attacks and strokes; or microvascular diseases such as neuropathy, nephropathy, and cancer [8]. In order to effectively reduce the incidence of T2DM worldwide and prevent concurrent infections, efforts must be undertaken to discern the etiology, to create rationale treatments for symptomatic patients, and to develop preventive measures.

### 1.2. Pathogenesis in T2DM

Diabetes complications resulting from an impaired glucose metabolism have been associated with retinopathy, nephropathy, and polyneuropathy [9]. When present, excess glucose is not oxidized and is shunted to the polyol pathway, consisting of two enzymes—aldose reductase (AR) and sorbitol dehydrogenase (SDH) [10]. AR reduces glucose to sorbitol in the presence of its co-factor, NADPH; SDH, with its co-factor nicotinamide adenine dinucleotide (NAD+), converts sorbitol to fructose—a more potent nonenzymatic glycation agent than glucose. Thus, an increase in glucose flux through the polyol pathway (as happens in T2DM) will decrease the available NADPH and increase the production of Advanced Glycation End products (AGE), leading to oxidative stress in a pathway we discuss later in this review [11]. The SDH oxidation of sorbitol to fructose also causes oxidative stress due to the co-factor conversion of NAD+ to NADH, the substrate for NADH oxidase which generates reactive oxygen species (ROS) [12]. Elevated sorbitol levels have also been associated with cellular and organ damage by directly depleting myoinositol (MI). This MI deficiency alters the metabolism of phosphoinositides and reduces diacylglycerol (DAG), inositol triphosphate (IP3) and protein kinase C (PKC) activation, resulting in a reduction in NA^+^/K^+^ ATPase pump activity. Metabolic pathway derangement causes conductance abnormalities, resulting in neurological complications such as the neuropathy observed in diabetic patients [10]. Additionally, SDH enzyme deficiency in nervous tissues, the kidney, and the lens and retina of the eye lead to the elevation of sorbitol, leading to the development and progression of retinopathy, cataracts and neuropathy observed in diabetic patients [13].

Concomitantly, the cellular antioxidant capacity of Glutathione (GSH)is diminished due to AR activity, which depletes the NADPH required for glutathione reductase (GSR) to recycle glutathione (GSH). A reduction in available NADPH, due to the overutilization of the Polyol Pathway, significantly decreases GSH levels and thus limits the ROS-scavenging activity of GSH, and further increases ROS levels [14]. A diabetic mouse model (MKR) vs. control study performed in 2010 revealed sorbitol levels 2.5 times higher in MKR when compared to a control Also found was 1.7 times lower levels of the reduced glutathione (rGSH) in skeletal muscle [15]. Therefore, it is understood that the overutilization of the polyol pathway due to glucose excess is a major source of oxidative stress, by limiting the recycling of GSH via GSR induced by diabetes.

### 1.3. The Production of Glutathione in T2DM

GSH synthesis occurs intracellularly in a two-step enzymatic reaction. First, glutamine is joined with cysteine via the rate-limiting enzyme glutamine–cysteine ligase (GCLC) making γ–glutamyl cysteine. The second enzyme required for GSH synthesis is glutathione synthetase (GSS), which links γ–glutamyl cysteine to glycine to form the functional molecule of GSH. Once formed, GSH can perform its function by absorbing and detoxifying reactive oxygen species to prevent cellular damage. GSH can be found in vivo in two forms—reduced, functional glutathione (rGSH/GSH), and oxidized glutathione (GSSG). GSSG can be recycled to form rGSH/GSH via the enzyme GSR which, as previously stated, requires NADPH [16].

GSH levels have been shown to be significantly compromised in individuals with T2DM, due to a diminishment in the levels of NADPH causing the decreased ability of GSR to recycle GSSG to GSH. Compared to healthy individuals, people with T2DM have lower levels of GCLC, the rate-limiting catalytic unit in producing GSH [17]. These decreased amounts of GCLC correlated with increased amounts of transforming growth factor beta (TGF-β) in patients with T2DM [17]. Furthermore, decreased GCLC was accompanied by deceases in both GSS and gamma glutamyl transpeptidase (GGT). Both enzymes are crucial for the synthesis and import of GSH into the cell. The results show decreased overall levels of GSH in T2DM [17]. The rise in GSSG and decline of rGSH/GSH is due to the excess of free radicals or ROS being produced via the AGE/Receptor for Advanced Glycation End products (RAGE) pathway discussed in great detail below. When produced, rGSH can detoxify the ROS by accepting the electrons and combining two molecules of rGSH/GSH to make an oxidized form of glutathione GSSG.

GGT functions throughout the body as a transport molecule, enabling the breakdown of extracellular GSH. The cysteine formed is then transported into the cells for GSH biosynthesis. The mechanism by which TGF-β decreases these enzymes has not been fully elucidated; however, key components have been found. The Antioxidant Response Element (ARE) is a regulatory enhancer gene sequence, which upon activation induces the synthesis of basal and inducible GSH synthesizing enzymes. Bakin et el. found signal transduction proteins intracellularly that respond to TGF-β. Once TGF-β binds to its cellular receptor, a serine–threonine kinase, it auto-phosphorylates Smad2 and Smad3, which then heterodimerize with Smad4 and can translocate to the nucleus and upregulate or downregulate genes, such as GCLC and Glutamate cysteine ligase modifier protein (GCLM) [18]. It has also been shown that TGF-β, in addition to downregulating GCLC mRNA production, also decreases the half-life of GCLC, resulting in increasing ROS intracellularly. When TGF-β interacts with its cellular receptor, it causes an increase in caspase activity which begins the apoptosis cascade. Inducing apoptosis cleaves proteins intracellularly, including GCLC among many others [19]. With these enzymes decreased, it is recognized that people with T2DM produce a lower amount of functional GSH. Another important enzyme responsible for restoring the amount of GSH is GSR, which converts GSSG to GSH utilizing the cofactor NADPH. Contrary to the aforementioned enzymes, GSR is found in excess in patients with T2DM, which our group postulates is a compensatory mechanism to overcome GSH deficiency. However, with a deficiency in NADPH—a necessary co-factor for the activity of GSR—there is no restoration of GSH despite the increased levels of GSR. Lagman et al. also found that the amount of GSSG in T2DM was elevated in members with HbA1C over 8% [17]. They also found a five-fold decrease in Tumor Necrosis Factor alpha (TNF-α), and a two-fold decrease in IFN-γ. The study findings suggest that administering L-GSH will be more efficacious in patients with T2DM, than administering N-Acetyl Cysteine (NAC), a precursor for GSH, due to the aforementioned decreased enzymatic levels [17]. Despite abundant GSR, there are increased levels of GSSG and decreased GSH in T2DM, and this could very well be caused by the diminished levels of NADPH in lieu of polyol pathway utilization.

### 1.4. AGE-RAGE and GSH Deficiency in T2DM

The first AGE was discovered in the 1960s with the discovery of HbA1C. AGEs are destructive in many ways, but we will focus on one we believe to be particularly important for the function of the immune system. The binding of AGE with its counterpart Receptor for Advanced Glycation End products (RAGE) starts a cascade, increasing the generation of ROS and activation of Nuclear Factor-kappa-B (NF-*_k_*B) which produces proinflammatory molecules interleukin-1 (IL-1) and interleukin-6 (IL-6). On top of the AGE-RAGE component with excess glucose in the cell, DAG is formed, which can lead to the activation of PKC. The activation of PKC involves a plethora of actions, including the activation of NF-*_k_*B, further increasing IL-1 and IL-6, as well as TGF-β, which, as described previously, results in a decrease in GCLC. With the combination of TGF-β inactivating the rate-limiting step of GSH production, and with the increase in IL-1 and IL-6 there is exacerbated inflammation elevating the levels of ROS in all surrounding cells. Studies have shown that this change in the cytokine environment will lead to an altered immune response [20].

### 1.5. T2DM and Tuberculosis

In 2017, tuberculosis (TB) caused an estimated 1.6 million deaths and an incidence of 10 million cases of TB developed worldwide [21]. TB has been on the rise since the 1980s, in part due to the HIV pandemic, multidrug resistance, and increased numbers of highly susceptible individuals with suppressed/altered immune systems, such as those with T2DM [8]. Currently, one-quarter of the population of the world is latently infected with *M. tb* [21]. Individuals with latent tuberculosis Infection (LTBI) are not infectious; however, active TB can occur due to the reactivation of a dormant infection as a consequence of granuloma liquefaction in immunodeficient or immunocompromised individuals, such as individuals with HIV or T2DM [5]. Several studies have looked at the correlation between the prevalence of T2DM and TB. In 2012, it was found that 50% of patients studied who tested positive for *M. tb* infection had either diabetes or pre-diabetes. Other studies which found similar accounts of diabetes and TB in India found that, in Mexico, 35% of patients testing positive for *M. tb* infection had diabetes. Furthermore, in 2011, an article found that with uncontrolled diabetes and TB infection there were more cavitary lesions, and a higher incidence of positive sputum cultures, even two months after initiation treatment. Thus, careful monitoring of the progression of *M. tb* infection and glucose control should be top priorities of physicians with patients with both illnesses [22]. According to the CDC, 20% of patients with TB have diabetes in the US.

**Figure 1:**
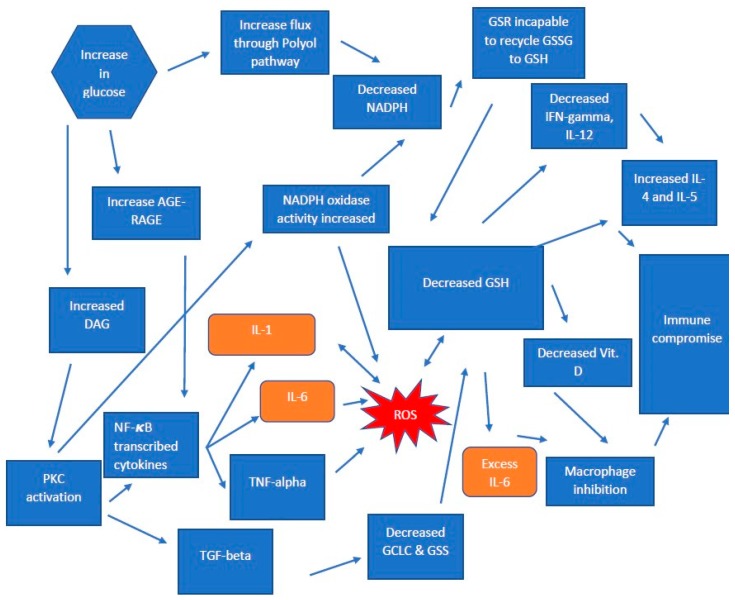
For oxidative stress and glutathione (GSH) deficiency in individuals with Type 2 Diabetes Mellitus (T2DM).

In patients with T2DM, the depletion of GSH is extremely prevalent, which may result in additional pathogenesis. Discussed later are the effects of GSH depletion in altering levels of cytokine production and the immune consequence. One cytokine that is extremely important for restricting and containing *M. tb* infection is TNF-α. This cytokine aids in the formation of granuloma to prevent the spread and wall off the pathogen. It has been shown that, in the absence of TNF-α, there will be an impairment in the formation of the granuloma, leading to the dissemination of the pathogen [23]. IFN-γ, another cytokine responsible for augmenting the effector functions of macrophages, was found to be decreased in patients with T2DM [17]. In a systematic review, patients with diabetes who were infected with *M. tb* had a risk ratio of 1.69 for treatment failure and death. The study also found an increased risk of relapse in patients with diabetes vs. non-diabetic individuals. The conclusion of the study found that improved glucose control and close monitoring in patients with the comorbidity of *M. tb* and Diabetes should be implemented to assist in the recovery process [23]. *M. tb* infection in healthy subjects will result in robust immune responses mediated by the competent immune system resulting in the formation of granuloma, which suppresses the spread of the pathogen. However, in patients with weakened immune systems, a reactivation of *M. tb* occurring in those with LTBI or primary infection can lead to active TB. We were able to find links between cytokine imbalance, as a consequence of diminished GSH levels due to decreases in the level of GSH synthesis and ability of the recycling enzymes. Increased TGF-β can be a major contributing factor to the decreasing levels of GSH synthesis enzymes. The inability of GSR in diabetic patients to restore in order to recycle GSSG to GSH is due to the depletion of NADPH by the Polyol pathway. With all these factors, it forms a sort of circle of perpetuation. (Visualized in Figure 1) *M. tb* has been a successful pathogen capable of evading the immune system, and those with a compromised immune system are at increased risk for both the reactivation of LTBI and development of active TB after initial exposure. While *M. tb* is not the only pathogen to cause an active disease in individuals with diabetes, we focused mainly on this pathogen due to the long-standing prevalence, as well as the global burden, of TB. Two cytokines—IFN- γ and TNF- α —which are imperative to the control and prevention of the spread of *M. tb*, respectively, are significantly compromised in individuals with T2DM (discussed below in the next section). We therefore believe that cytokine dysregulation in individuals with T2DM causes impaired immune responses against *M. tb*, resulting in active TB.

### 1.6. Cytokine Production and Immune Responses against M. tb Infection

Efficient communication between cells is necessary to modulate an adequate immune response in individuals for cell migration and specific instruction. This is a critical role for cytokines and chemokines. Cytokines are small, soluble proteins that are produced by the host immune cells which influence the activity of other cells by acting in a paracrine manner [24]. Different immune cells, such as macrophages and neutrophils, initiate cytokine production when interacting with a pathogen. Multiple pattern recognition receptors (PRR) on these cells recognize various bacterial factors, such as mycobacterial cell wall components, secreted molecules, and nucleic acids derived from mycobacteria, which promote cytokine production [19,20]. The cytokines discharged by these cells have essential regulatory properties, and contribute to the host defense against *M. tb* through the formation of a granuloma which leads to the containment and extermination of bacteria [25,26]. Granulomas are composed of macrophages, multinucleated giant cells, CD4+ and CD8+ T-cells, B-cells and neutrophils [27]. Within an immunocompetent individual, the interaction between *M. tb* and granulomatous cells of the immune system results in the constant secretion of cytokines; particularly, TNF-α, (formation of the granuloma), interleukin-10 (IL-10)(immune modulation), IL-6 (inflammatory cytokine), interleukin-2 (IL-2)(T-cell growth factor), interleukin-12 (IL-12)(stimulation of cells to produce IFN-γ), and IFN-γ (macrophage stimulation) [28].

TNF-α secreted by macrophages, dendritic cells (DC), and T-cells augments effector immune responses against *M. tb* infection [26,29]. Additionally, upon infection, pulmonary epithelium and lung fibroblasts produce chemokine (C-X-C motif) ligand 8 (CXCL8), which promotes the rapid recruitment of neutrophils [30]. It was previously shown, that after the ingestion of dead or dying *M. tb*-infected apoptotic neutrophils at the infection site, a proinflammatory response is triggered and macrophages become activated, releasing TNF-α [31]. Thus, it was demonstrated that TNF-α promotes the production of chemokines and chemokine receptors, and also an expression of adhesion molecules, which affect the formation of granulomas in *M. tb*-infected tissues [32]. TNF-α also works in synergy with IFN-γ to induce nitric oxide production [33].

Another important modulator of the immune response is the neutrophil-mediated release of IL-10, which is known to decrease inflammation. IL-10 is produced by phagocytes of lung lesions and reduces expression of TNF-α and IL-12 [34]. IL-10 also blocks phagosome maturation and allows the pathogen to survive in alveolar macrophages, and through reduction of T-cell responses, it may affect the integrity of the granulomas, thus promoting the transmission of *M. tb* by predisposing the host to lung cavitation [35]. IL-10 possesses macrophage-deactivating properties and reduces IL-12 production, which in turn decreases IFN-γ production by T-cells [36]. These findings indicate that IL-10 plays an anti-inflammatory role and may counter the macrophage-activating properties of IFN-γ. Therefore, the balance between TNF-α with antagonistic IL-10 and synergistic IFN-γ cytokines plays a role in fine-tuning the tissue damage in the granulomas, as well as its bactericidal effects [37].

When *M. tb* or apoptotic macrophages and neutrophils containing *M. tb* interact with DCs, it causes the DCs to mature and become able to stimulate T-cells through the expression of major histocompatibility complex (MHC) and co-stimulatory molecules, with the aid of the secretion of IL-12 and IFN-γ [38]. IL-12 inducers can polarize CD4+ T-cells toward a T-helper (T_H_1) phenotype [39]. T_H_1cells produce IFN-γ, IL-2, IL-12 and TNF-α—cytokines important for the suppression of *M. tb* growth and replication [40]. On the other hand, Type-2 T-helper phenotype (T_H_2) responses lead to the production of IL-4, IL-5, and IL-10, which exert anti-inflammatory effects [41].

CD4+ and CD8+ T-cells are important in maintaining specific immunity against *M. tb*, and protective immunity is associated with antigen presentation by DC to T-cells [42]. Although MHC class II restricted CD4+ T-cells play an important role in protection against *M. tb*, MHC Class I restricted T-cells act later in the infectious cycle by killing *M. tb*-infected cells through the secretion of cytotoxic molecules, such as perforin, granzymes, and granulysin [43].

IL-2, also called T-cell growth factor, is produced by T_H_1 cells. IL-2 is responsible for maintaining the CD4 and CD8 T-cell viability, and thereby amplifies T-cell responses against *M. tb* infection [37].

IL-6 is another cytokine involved in the immune response against *M. tb* which has multiple roles, including inflammation and T-cell differentiation [44]. During infection, IL-6 and TGF-β control the relative levels of expression of the transcription factor FoxP3. These T-regs have multiple inhibitory effects, and by limiting the intensity of the T-cell response to *M. tb*, they attempt to balance potentially harmful immune responses [38,39]. IL-17 mediates the cellular recruitment of neutrophils via CXCL8 in order to form lung lymphoid follicles, providing optimal macrophage activation and bacterial control during *M. tb.* infection. However, the hyperactivity of T_H_17 cells can lead to an increased pathological state through the IL-17 mediated influx of neutrophils, which can cause tissue damage [41,42].

TB is a complex disease, and as such, the role of any particular cytokine cannot be categorized so easily as either “beneficial” or “harmful”. However, based on the amount produced and the circumstances, these cytokines can cause either defensive or pathologic effects, by either working synergistically or antagonistically to the immune system in order to control the infection.

### 1.7. Why GSH Is Important in a Functioning Immune System

In 2003, Venketaraman et al. established the importance of GSH in the immune response controlling the infection of *M. tb* [45]. GSH levels were found to be decreased in red blood cells and peripheral blood mononuclear cells isolated from patients with active pulmonary TB. Dr. Venketaraman’s group also found that the administration of a precursor of GSH—N-acetyl cysteine (NAC)—to isolated immune cells improved the control of *M. tb*, as well as decreased the levels of pro-inflammatory cytokines such as IL-6, TNF-α and IL-1 [45]. Furthermore, through its role as a Nitric Oxide (NO) carrier, the addition of S-Nitrosoglutathione (GSNO) to *M*. *tb* cultures was found to be mycobactericidal [46]. Moreover, the addition of NAC, along with cytokines IL-2, and IL-12 caused a significant enhancement in the effector functions of natural killer (NK) cells, which in turn caused an increased production of IFN-γ, a macrophage-activating cytokine [44,47,48,49]. The immune system requires different cytokines present to mount the proper responses. The T_H_1 response, for example, which requires IL-12 and IFN-γ produced by the T_H_1 cells, can further activate macrophage effector response [50]. With the infection present, ROS are produced to assist in the killing of bacteria; however, they can be harmful to the host system if not absorbed via GSH and other antioxidants to prevent cellular damage. It has been shown that ROS and GSH levels can alter the cytokine release of T-cells [51]. Depletion of GSH due to ROS production will lead to NF-*_k_*B binding to the DNA, resulting in the production of IL-1, IL-6 and TNF-α. In contrast, GSH repletion will suppress the production of the aforementioned cytokines [51,52,53]. With a depletion of GSH during infection or T2DM, this could cause NF-*_k_*B release and upregulate the production of IL-1, TNF-α, and IL-6—all inflammatory cytokines. Although IL-6 is necessary for proper macrophage activity, Van Heyningen’s group found that the overproduction of IL-6 can lead to macrophages’ inability to respond properly to the infection [48]. With this being said, TNF-α is an extremely important cytokine for the formation of granulomas in *M. tb* infection. A cytokine cannot be classified as “beneficial” or “detrimental”; rather, in order to produce intended effect it should be in the right site at the right time, but, as stated previously, overproduction can cause disarray, desensitization and malfunction. With each of these studies, it should now be apparent that GSH is extremely important for the proper function of the immune system, whether assisting as an NO carrier, as GSNO to assist in the killing of the infection, or scavenging excess ROS to control cytokine levels to mount a proper response. We believe the augmented immune system in diabetic patients is primarily due to the decrease in GSH, which leads to a depletion in IL-12. Deficiency of this cytokine has been shown in several studies to lead to recurrent infections, while the addition of NAC or L-GSH restored the IL-12 and IFN-γ, which in turn enhanced mycobacterial killing [44,47,54,55,56]. Studies have also found that when there is an increase in IL-4 and IL-5, the cytokines responsible for polarizing the helper T-cells to a T_H_2 response [57,58]. Thus, further proving that the levels of ROS or GSH are a crucial deciding factor when creating an immune response.

### 1.8. Vitamin D and Macrophage Activation

Vitamin D is a secosteroid which can be ingested (vitamin D3 and vitamin D2) into the body or endogenously produced in the skin upon sun exposure (vitamin D3) [59,60]. Vitamin D has displayed anti-inflammatory effects, as supplementation with vitamin D results in decreased radical oxygen species (ROS) and pro-inflammatory cytokines [61,62].

GSH plays a role in maintaining vitamin D regulatory genes; GSH deficiency hinders the expression of vitamin D-binding proteins and receptors [63,64]. Furthermore, supplementation with L-cystine, a precursor for GSH, increases levels of vitamin D and its binding proteins [63,65].

Thus, we postulate that a decrease in GSH indirectly affects the immune system via decreasing the concentrations of vitamin D. However, further studies are needed to confirm at what hemoglobin A1c the deficiency of Vitamin D—due to decreases in GSH—occurs. The mechanism underlying this phenomenon can be attributed to the role of vitamin D in adaptive immunity [64]. Vitamin D assists macrophages to inhibit the proliferation of *M. tb* [59,60,66]. *M. tb* binds to the toll-like receptors seen on the cell surface of macrophages, upon which the expression of the vitamin D receptors and 1-alpha hydroxylase is upregulated [67]. The activated vitamin D then induces the transcription of human cathelicidin (hCAP18) and beta-defensin 4 (DEFB4), both of which are crucial to antimycobacterial function including the auto-lysosomal elimination of *M. tb* [59,66,67,68,69]. The importance of this intracrine pathway was demonstrated by Zhao’s study in which the cellular ability to eradicate *M. tb* in DM patients improved upon vitamin D supplementation [65].

## 2. Summary

It has been shown in previous studies, that an excess of glucose in systemic circulation can cause an increase in ROS and the proinflammatory cytokines IL-1 and IL-6, which at high enough concentrations can inhibit the function of macrophages. Due to excess glucose, TGF-β becomes increased through the activation of AGE and its receptor RAGE, through PKC, which inhibits the manufacturing of GSH. The oxidized form of glutathione—GSSG—can be recycled if the body has enough NADPH utilizing the enzyme GSR. However, in diabetic patients, NADPH is being utilized heavily on the polyol pathway, leading to an overall decrease in GSH levels and an increase in ROS, which can alter the cytokine levels in the body [55]. GSH increase has been shown to upregulate the production of IFN-γ and IL-12, favoring the host immune response against *M. tb* infection.

The peripheral neuropathy and altered immune system can be traced back to a common metabolic pathway, but for different reasons. One of the complications of T2DM, peripheral neuropathy, was linked to the Polyol pathway and the build-up of sorbitol due to an SDH deficiency in the nervous tissue. The complications involving the immune system are not due to products from the Polyol pathway but rather from the overutilization of NADPH. The depletion of NADPH leads to the inability of GSR to recycle GSH, causing decreased GSH, increased ROS, differing cytokine profiles and, ultimately, immune system compromise.
